# Panophthalmitis in a case of Glanzmann thrombasthenia – The dilemma in diagnosis

**DOI:** 10.22336/rjo.2025.65

**Published:** 2025

**Authors:** Lahari Velivelli, Thanuja Gopal Pradeep, Sandeep Sreerama Reddy, Rashmi Palassery

**Affiliations:** 1 Department of Ophthalmology, Ramaiah University of Applied Sciences, Bangalore, India; 2 Department of Ophthalmology, Ramaiah Medical College, Ramaiah University of Applied Sciences, Bangalore, India; 3 Department of Infectious Diseases, Ramaiah Medical College, Ramaiah University of Applied Sciences, Bangalore, India; 4 Department of Hemato-oncology, Ramaiah Medical College, Ramaiah University of Applied Sciences, Bangalore, India

**Keywords:** Glanzmann thrombasthenia, panophthalmitis, post blood transfusion, fortified insulin eye drops, phthisis bulbi, endophthalmitis, GP = Glycoprotein, CT = Computed Tomography, MRI = Magnetic Resonance Imaging, B-Scan = Bi modal scan, HLA = Human Leucocyte Antigen

## Abstract

We present a rare case of a middle-aged female diagnosed with Glanzmann Thrombasthenia who developed panophthalmitis following platelet transfusion. The patient presented with acute vision loss, proptosis, and ocular pain in the right eye following a platelet transfusion. Imaging confirmed panophthalmitis, while cultures were inconclusive. The patient was treated with intravenous and fortified topical antibiotics along with platelet transfusions. Despite aggressive management, the right eye progressed to phthisis bulbi. Systemic infection was controlled, and ocular pain and swelling significantly reduced. The patient was managed with systemic and fortified topical antibiotics under the care of a multidisciplinary team, including ophthalmologists, hematologists, and infectious disease specialists. This report highlights the diagnostic challenges, management dilemmas, and the importance of an interdisciplinary approach in managing infectious complications in patients with inherited platelet function disorders.

## Introduction

Glanzmann Thrombasthenia is a rare, inherited autosomal recessive disorder characterized by defective platelet aggregation due to abnormalities or deficiencies in the glycoprotein IIb/IIIa complex on platelet membranes . This results in impaired clot formation, leading to prolonged bleeding episodes. The condition is typically diagnosed in early childhood, with patients often requiring recurrent platelet transfusions to manage bleeding episodes. The only treatment available is allogeneic hematopoietic stem cell transplantation. While bleeding-related complications, such as mucocutaneous hemorrhage, recurrent hyphema, choroidal hemorrhage, and conjunctival bleeding, have been well-documented , ocular manifestations are exceedingly rare.

Panophthalmitis, a severe, sight-threatening infection involving all layers of the eye and adjacent orbital structures, is an uncommon complication in patients with Glanzmann. Infections following blood product transfusions, though rare, have been reported in other conditions, such as dengue hemorrhagic fever. However, to our knowledge, there are limited reports of panophthalmitis occurring post-platelet transfusion in these patients.

## Case report

A middle-aged female patient, diagnosed with Glanzmann Thrombasthenia at the age of two, underwent regular platelet and occasional packed red blood cell transfusions at approximately every six months. Although bone marrow transplantation was offered as a curative option, it was deferred by the family for unknown reasons.

The patient presented to the emergency department with complaints of sudden diminution of vision in the right eye following a platelet transfusion administered one day prior. She reported experiencing fainting episodes on the evening of the transfusion, followed by the onset of fever and decreased vision in the right eye. The vision loss progressed rapidly, accompanied by severe ocular pain and redness, prompting her to seek emergency care.

**Fig. 1:**
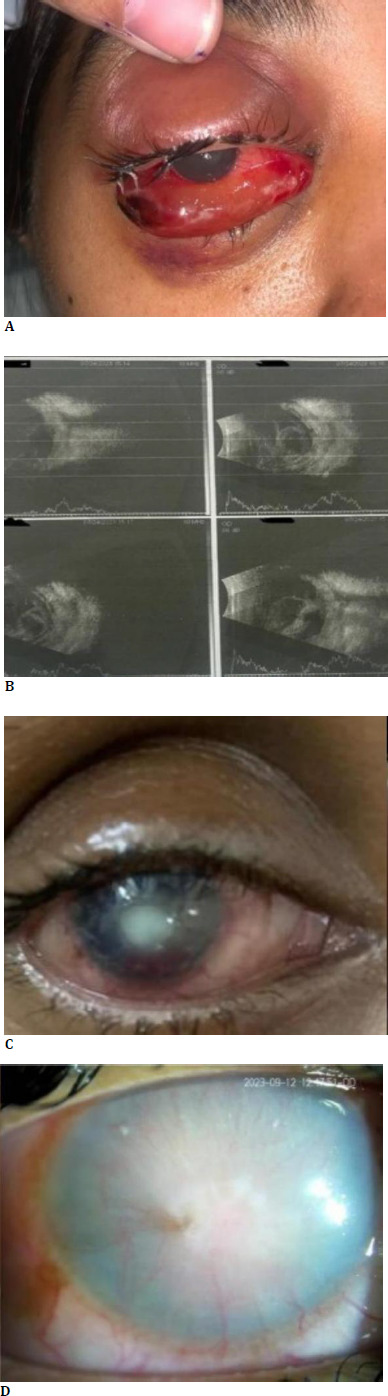
**A** Image of the Right eye showing proptosis and chemosis; **B**. B scan showing hyperintense echoes in the vitreous with retinochoroidal thickening suggestive of panophthalmitis; **C**. Non-healing persistent epithelial defect; **D**. Healed corneal ulcer at the end of 2 months

On examination, the visual acuity in the right eye was limited to perception of light, but the light projection was inaccurate (**Fig.1A**). Extraocular movements were restricted in the right eye. Clinical evaluation revealed periorbital edema, conjunctival chemosis, and proptosis. The cornea appeared hazy with stromal edema, the anterior chamber was shallow, and intraocular pressure was elevated. The left eye exhibited normal anterior and posterior segment findings with a visual acuity of 6/12 on Snellen testing. The differential diagnosis at this point was panophthalmitis and carotid cavernous fistula with a history of trauma. MRI scan of the orbit and brain showed the altered signal intensity of the right cornea and sclera with internal debris within the vitreous, suggestive of panophthalmitis. B-scan ultrasound of the right eye revealed increased retinal choroidal (RC) thickness with a central hyperechoic mass in front of the optic nerve (**Fig. 1B**). A CT Angiogram with CT-Brain was done, and it showed no evidence of carotid-cavernous fistula.

The patient was started on fortified tobramycin eye drops and fortified ceftazidime eye drops to cover both gram-negative and gram-positive organisms, along with intravenous meropenem and vancomycin in consultation with an infectious disease specialist. The patient also received platelet transfusions to stabilize her general condition.

On follow-up, vision in the right eye was negative for perception of light, and the globe showed phthisis changes with altered globe contour. Extraocular movements were complete and painless. Chemosis and proptosis were reduced, and the cornea showed deep vascularization with a punched-out corneal ulcer (**Fig. 1C**). The patient had a persistent epithelial defect for 3 weeks after stopping all antibiotics and increasing the artificial tears. Moreover, the patient was started on insulin eye drops (0.25 ml of Human Actarapid was mixed with carboxymethylcellulose eye drops). The epithelial defect healed in 1 week. Left eye vision was 6/6 (aided).

## Discussion

There are a few reported cases of ocular manifestations in Glanzmann Thrombasthenia, and all are related to bleeding complications. Dinakaran et al. reported a case of conjunctival bleeding following topical phacoemulsification in a 79-year-old woman diagnosed with acquired thrombasthenia [2]. Saatchi et al. described a case of massive choroidal hemorrhage with uncontrollable intraoperative bleeding during vitrectomy [3]. Komboroglu et al. documented recurrent hyphema in a 12-year-old boy following blunt ocular trauma, which resulted in vision loss due to corneal blood staining and vitreous hemorrhage [4]. *Tan et al*. highlighted the rare occurrence of *Endogenous Serratia marcescens* endophthalmitis, emphasizing the potential for hematogenous bacterial spread to the eye post-transfusion, underlining the need for careful monitoring for complications in patients with Glanzmann Thrombasthenia [5]. Ravani et al. stated that cases of panophthalmitis and endophthalmitis are rare but serious complications following platelet transfusion in patients with dengue hemorrhagic fever, highlighting the need for careful monitoring and infection control [6].

In our case, the patient had a confirmed diagnosis of Glanzmann Thrombasthenia and presented with acute vision loss and elevated intraocular pressure following a platelet transfusion. The patient also reported fainting episodes after the transfusion. The differential diagnoses included panophthalmitis, retro-orbital hemorrhage, and carotid-cavernous fistula. A CT angiogram was performed to rule out carotid-cavernous fistula and retro-orbital bleeding, both of which were excluded. MRI of the orbit and brain revealed altered signal intensity in the right cornea and sclera, along with internal vitreous debris. A B-scan showed increased retinal choroidal thickness findings suggestive of panophthalmitis, thereby confirming our diagnosis.

## Conclusion

This case highlights the diagnostic and therapeutic challenges associated with managing infectious complications medically, whereas significant bleeding risks complicate surgical interventions. Additionally, this report underlines the importance of a multidisciplinary approach of specialists in optimizing patient outcomes.
